# *PmCBFs* synthetically affect *PmDAM6* by alternative promoter binding and protein complexes towards the dormancy of bud for *Prunus mume*

**DOI:** 10.1038/s41598-018-22537-w

**Published:** 2018-03-14

**Authors:** Kai Zhao, Yuzhen Zhou, Sagheer Ahmad, Xue Yong, Xuehua Xie, Yu Han, Yushu Li, Lidan Sun, Qixiang Zhang

**Affiliations:** 1 Beijing Key Laboratory of Ornamental Plants Germplasm Innovation & Molecular Breeding, National Engineering Research Center for Floriculture, Beijing Laboratory of Urban and Rural Ecological Environment, Key Laboratory of Genetics and Breeding in Forest Trees and Ornamental Plants of Ministry of Education, School of Landscape Architecture, Beijing Forestry University, 100083 Beijing, China; 2 Beijing Advanced Innovation Center for Tree Breeding by Molecular Design, Beijing Forestry University, 100083 Beijing, China

## Abstract

The survival in freezing temperature for woody plants is exclusively dependent on the perception of coldness and induction of dormancy. CBF/DREB1 transcriptional factors join cold-response conduits and the *DAM* genes, especially *PmDAM6*, are well-known regulators of dormancy. Despite the immense importance, little is documented on the association between CBF proteins and the complexity of the promoter region in *PmDAM6* with the function of bud dormancy in *P*. *mume*. Therefore, this study was based on the cloning of *PmDAM6* and six *PmCBFs* to evaluate their integral roles in the process of bud development. The consistency of expressions in either vegetative or reproductive buds provided a negative control from *PmCBFs* to *PmDAM6* during the onset of dormancy. Besides, PmCBF5 could form heteromeric complexes with PmDAM1 and PmDAM6. PmCBF1, PmCBF3, and PmDAM4 recognized the promoter of *PmDAM6* by the alternative binding sites. Therefore, the interactions of these genes formulated the base of an obvious model to respond to the coldness and engendered dormancy release. Findings of this study will further help the unveil the genetic control of bud dormancy and its augmentation in *P*. *mume* and may offer an explanation for the vernalization.

## Introduction

Formation of a bud for perennial plants is often concomitant with its ability to enter dormant state^1^ and prevailing cold climates challenge the buds in their capacity to retain the reproductive potential unless favorable conditions arrive^2^. Therefore, dormancy helps plants to escape bad environmental circumstances and to keep their growth potential alive^3^. *Prunus* genus is rich in fruit-bearing species like peach (*Prunus persica*), plum (*Prunus domestica*), apricot (*Prunus armeniaca*), Japanese apricot (*Prunus mume*), almond (*Prunus dulcis*) and cherry (*Prunus avium*). These species are capable of mending their growth habits in accordance with seasonal variations. *P*. *mume* has been cultivated in China for over 3000 years. The flower of this tree can bloom in low temperature, earlier than many other species in *Prunus*. However, the bud induction and floral organ differentiation appeared in the next year. Therefore, it is of immense importance to search out the genetic factors underlying the coldness perception and induction of dormancy^4^. According to whole genome sequencing analysis, six *PmDAM* genes were identified, their tandem repeats were distributed in the genome, and six CBF binding sites were found in the upstream of *PmDAM* genes^5^. It is supposed that the *PmDAMs* and their CBF binding sites may be the key factors controlling early dormancy release^6^.

MADS-box gene family contains transcriptional factors which were found important to have applications in plant organogenesis covering flower organ development, determination of meristematic identity and the vegetative transformation into reproductive phase^7^. Dorman cy associated MADS-box (*DAM*) genes play integral roles in specifying the dormancy transitions during the growth curves^8–11^. In *P*. *persica*, the expression profiles of *DAM* genes are associated with obvious seasonal changes of temperature^12,13^. As for *P*. *persica* and *P*.*avium*, the expressions of *DAM* genes are related to the quantification of the cold response and flowering date manipulation under varying environmental circumstances^13–15^. *PpDAM5* and *PpDAM6*, showing the expressions closely related to cooling capacity and flowering date^14,16^, can inhibit peach bud growth at low temperature^13^. The expressions of *PpDAM5* and *PpDAM6* were up-regulated during internal dormancy, but down-regulated during dormancy release^3,17^.

Low temperature significantly affects C-Repeat Binding Factor (*CBF*), a cold response signal factor isolated by Stockinger^18^. In *Arabidopsis*, there are three *CBF/DREB1* genes guiding the signal pathway to low temperature response^19^. *CBFs* can function to enhance the cold resistance of plants by inducing the downstream genes^20,21^. Benedict^22^ transferred the *AtCBF1* gene into poplar, comparing it to the wild-type poplar, and found that the cold resistance of the transgenetic poplar was highly enhanced. *CBF* genes in flowering peach can increase the cold tolerance and can cause dormancy in short-day conditions^23^. When a peach CBF gene was overexpressed in apple, both non-acclimated and acclimated freezing tolerance was observed and displayed these phenotypes^24^.

For many woody plants, *DAM* genes are involved in plant dormancy induction, and *CBF* genes are related to cold-response pathway. On account of the participation of these genes in supervising the plant growth and development in cold climate, it is necessary to investigate their functions in plant growth and dormancy control. Therefore, studying the interactive influence of both the gene types as determinants of bud activity in *P*. *mume* could be a nascent concept inviting plentiful directions for future studies. In this research, we cloned *PmDAM6* and six *PmCBFs*, ascertained their expression profiles in flower bud, flower, leaf bud, and leaf, and tested their roles in the simulated bud dormancy. In addition, multiple interaction experiments were carried out to investigate the protein-protein and protein-DNA associations among PmCBFs and PmDAM6 in controlling bud growth and dormancy. This will invite future studies on molecular perceptive of bud control and cold response conduit.

## Results

### PmCBFs bind to the promoter of *PmDAM6*

Based on the research of *P*. *mume* genome, there exist more binding sites (C-repeat/DRE element) in the promoter of *PmDAM6* than those of other *PmDAMs*. In this research, we obtain the upstream fragment of *PmDAM6*. Indeed, there are three potential binding sites in the 1 kb region of *PmDAM6*’s promoter, and four in the 2 kb region. To uncover the role of *PmCBFs* and *PmDAM6* in induction and release of flower bud dormancy, we conducted yeast one hybrid (Y1H) experiments to explore the regulation between six PmCBFs and the promoter of *PmDAM6*.

**Figure 1:**
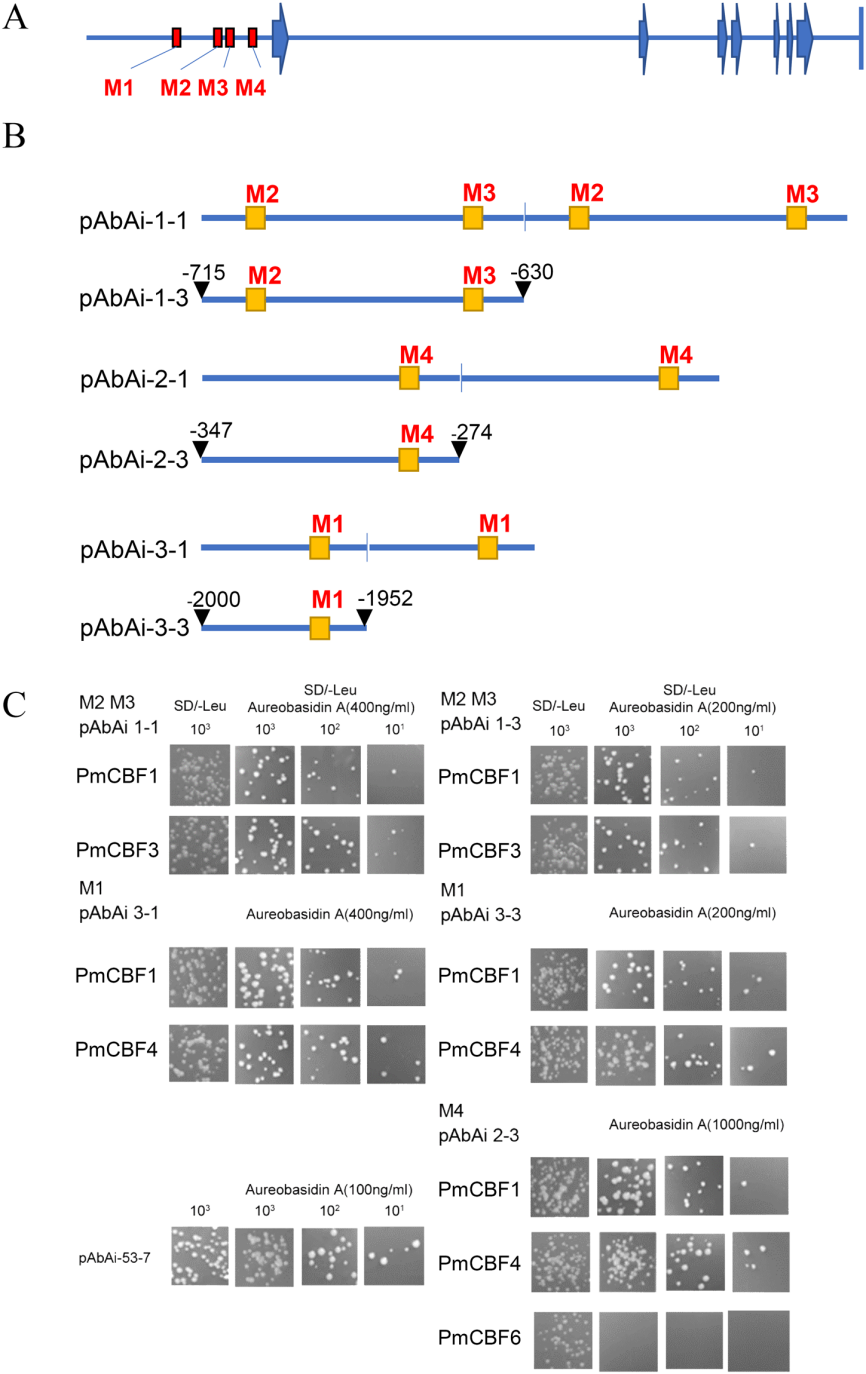
PmCBFs bind the promoter of *PmDAM6 in vivo*. (**A**) The genomic structure and promoters of *PmDAM6* and the CBF-binding sites in were marked by red blocks. (**B**) The designed baits in Y1H, the whole promoter was split into three part about 100 bp. (**C**) The results of Y1H assays between PmCBFs and the baits.

As shown in Fig. 1A, the promoter of *PmDAM6* (Supplementary Data S2) was separated into four potential binding sites based on the position of CCGAC: M1, −1971; M2, −706; M3, −648; M4, −294. Then, baits (length less than 100 bp) were designed as shown in Fig. 1B. A total of six bait vectors were constructed, M2 and M3 were designed in one bait, which got close to each other, the other two, M1 and M4, were independently performed as baits, then the original sequences of these three baits were duplicated respectively to form other three baits (Supplementary Data S3). Five of these six baits were tested negative to perform the following yeast one hybrid. The corresponding working concentrations of Aureobasidin A (AbA) were as follow: pAbAi-1-1, 400 ng/ml; pAbAi-1-3, 200 ng/ml; pAbAi-2-3, 1000 ng/ml; pAbAi-3-1, 400 ng/ml; pAbAi-3-3, 200 ng/ml. And the pAbAi-2-1 was not considered as a bait because of its normally growing under1000 ng/ml AbA.

All yeasts with successful transformations showed normal growth on the SD/-Leu solid medium (Fig. 1C). The Y1H assays showed that PmCBF1 and PmCBF3 could associate with baits of pAbAi-2-3, pAbAi-3-1, and pAbAi-3-3; and PmCBF1 and PmCBF4 could bind to baits of pAbAi-2-3, pAbAi-3-1, and pAbAi-3-3. These results suggested that PmCBF1 and PmCBF3 recognized the promoter of *PmDAM6* by the binding site M2 and M3, PmCBF1 and PmCBF4 could discern M1 and M4. However, PmCBF2, PmCBF5, and PmCBF6 failed to bind to the four C-repeat/DRE sites of *PmDAM6* promoter.

### *In vivo* protein-protein interactions among PmCBFs and PmDAM6

**Figure 2:**
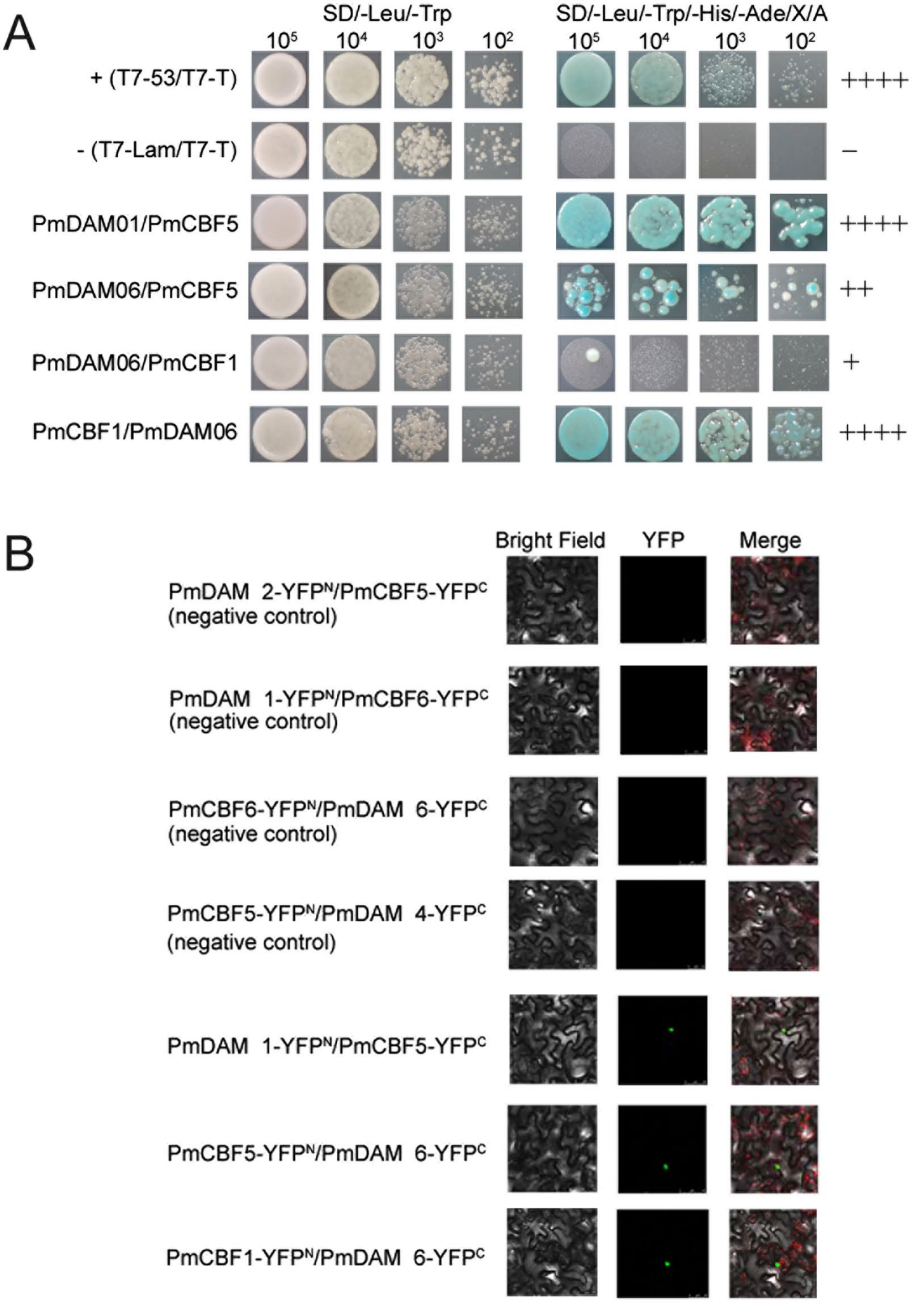
Protein-protein interactions between PmCBFs and PmDAMs. (**A**) Yeast two-hybrid analysis of the protein interactions between PmCBFs and PmDAMs. T7-53/T7-T was positive control, and T7-Lam/T7-T was negative control. The symbol (+) was represented the capacity of the reaction. The more numbers of the symbol (+), the more stronger capacity of the reaction. (**B**) BiFC analysis of the protein interactions between PmCBFs and PmDAMs. The green fluorescent presented protein position. The red fluorescent showed the chloroplast position.

Based on yeast two hybrid (Y2H) and bimolecular fluorescence complementation (BiFC) assay, a further exploration about PmCBFs and PmDAMs was made. According to Yeast two-hybrid experiments, PmCBF5 showed a strong interaction with PmDAM1 and a general interaction with PmDAM6 (Fig. 2A). Moreover, protein-protein interactions among PmDAM6 and PmCBFs were established by BiFC with a yellow fluorescent protein. Fluorescence released by these yellow fluorescent proteins was positioned at nucleus, suggesting the strong inclination among PmCBF5 and these two PmDAMs. PmCBF5 could form heteromeric complexes with PmDAM1 and PmDAM6 (Fig. 2B). As shown in Supplementary Fig. S3, there were no interactions in PmDAM1-YFP^N^/ YFP^C^, YFP^C^/ PmCBF5-YFP^N^, PmCBF5-YFP^C^/ YFP^N^, and YFP^N^/ PmDAM6-YFP^C^. In order to exclude the false positives, a member from the same protein family can be chosen to execute a negative control^25^. Therefore, the interactions of PmDAM2-YFP^N^/ PmCBF5-YFP^C^ and PmDAM1-YFP^N^/ PmCBF6-YFP^C^ were used as the negative controls of PmDAM1-YFP^N^/ PmCBF5-YFP^C^; the interactions of PmCBF6-YFP^N^/ PmDAM6-YFP^C^ and PmCBF5-YFP^N^/ PmDAM4-YFP^C^ were used as the negative controls of PmCBF5-YFP^N^/ PmDAM6 -YFP^C^. Fluorescent was not detected the in these negative tests.

### Crossover of the expressions between *PmCBFs* and *PmDAM6* in flower bud dormancy

**Figure 3:**
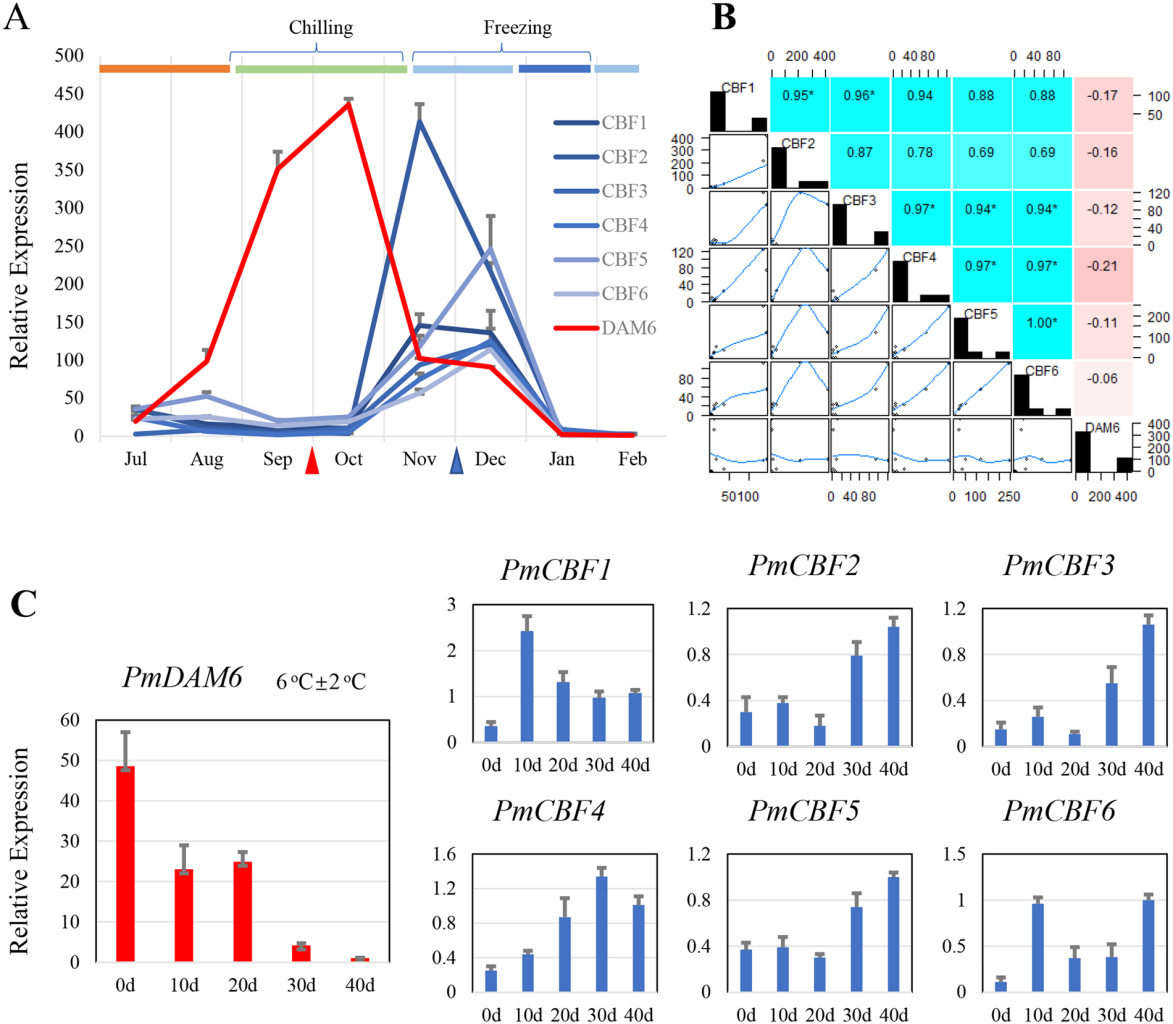
Expression patterns of *PmDAM6* and *PmCBFs* in flower buds of *P*. *mume*. (**A**) The expression levels of *PmCBFs* and *PmDAM6* in natural environment, the arrow denoted the sampling stages for simulated tests. (**B**) Correlations between *PmCBFs* and *PmDAM6*. The upper half matrix displayed the values of correlation coefficient, stars (*) marked the significant correlation members between two genes. The lower half display the linearity of the expression values. (**C**) The expression of *PmCBFs* and *PmDAM6* in simulated dormancy under low temperature from the flower bud, the samples were collected in the first stage (red arrow in A). (Warm: higher than 20 °C, Chilling: lower than 20 °C, Freezing: lower than 0 °C).

In Beijing, *P*. *mume* ‘SanlunYudie’ begins to form buds from June to July, undergoes flower bud differentiation from July to November, steps into dormancy from September to October, retains dormancy from November to January, and initiates dormancy breaking from January to February (Fig. 3A). From July to October, *PmDAM6* showed up-regulated in the chilling environment with a temperature under 20 °C and down-regulated in the coldness deepened weather. However, the expression tendencies of six *PmCBFs* during different periods of flower bud development were basically similar with high positive correlations (Fig. 3B) and were induced vastly under low temperature before freezing. The transcript levels of *PmCBFs* were low during July-October and January-February, but were high during November-December. The high expression of *CBF* genes seemed to inhibit the expressions of *DAM* genes. Together with all expression data, we can conclude that PmDAM6 and PmCBFs function in two temperature regions, respectively. This means PmDAM6 actively respond in the chilling temperature (below 20 ^o^C) and PmCBFs dominate in the freezing zone (below 0 °C), and the regulations in this biological process between these genes are continuous with a suppressive tendency.

### Expression patterns of *PmDAM*6 and *PmCBFs* in different blooming stages and different flower structures

**Figure 4:**
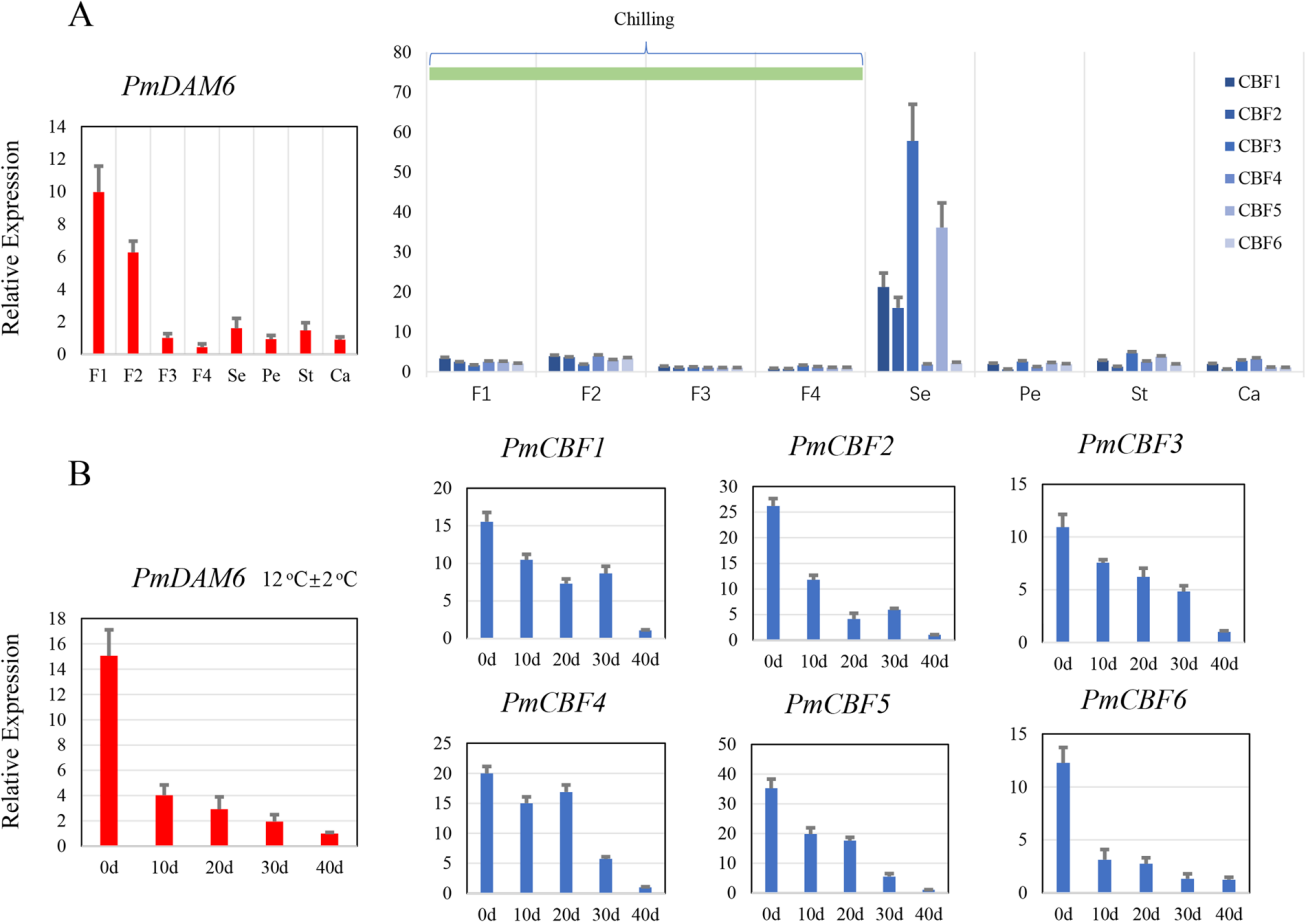
Expression patterns of *PmDAM6* and *PmCBFs* in bloom process and different flower structures. (**A**) The flower blooming process was manually divided into four stages according to the size of buds. F1:Small flower bud;F2:Big flower bud;F3:First blooming;F4:Full blooming. The mature flower was disassembled into four parts. Se:Sepal;Pe:Petal;St:Stamen;Ca:Carpel. (**B**) The expression levels of *PmDAM6* and *PmCBFs* in the simulated dormancy release test, the samples were collected from the second stage (blue arrow in Fig. 3A). (Warm: higher than 20 °C, Chilling: lower than 20 °C, Freezing: lower than 0 °C).

When the temperatures return to chilling, the flower bud started to grow and bloom. To explore the expression patterns of *PmDAM6* and *PmCBFs* genes in the different blooming stages and different floral structures, the blooming process was divided into four stages. On the whole, the expression trends of *PmDAM6* also declined from F1 to F4. In addition, *PmDAM6* were expressed in all the floral structures, and the expression of *PmCBFs* kept low levels and got lower in the flower blooming process (Fig. 4A). The expression tendencies of the *PmCBF*s in the flower blooming stages were basically the same, the transcript levels were relatively high in F1 and F2, but low in F3 and F4. According to the expression patterns of *PmCBF*s in different flower structures, *PmCBF*s were divided into two groups. *PmCBF1*-4 showed high expression specificity in sepals. The other type genes, including *PmCBF*5*-6* were expressed in all of the floral structures without significant differences.

### Expression patterns of *PmDAM*6 and *PmCBFs* in leaf buds and leaves

**Figure 5:**
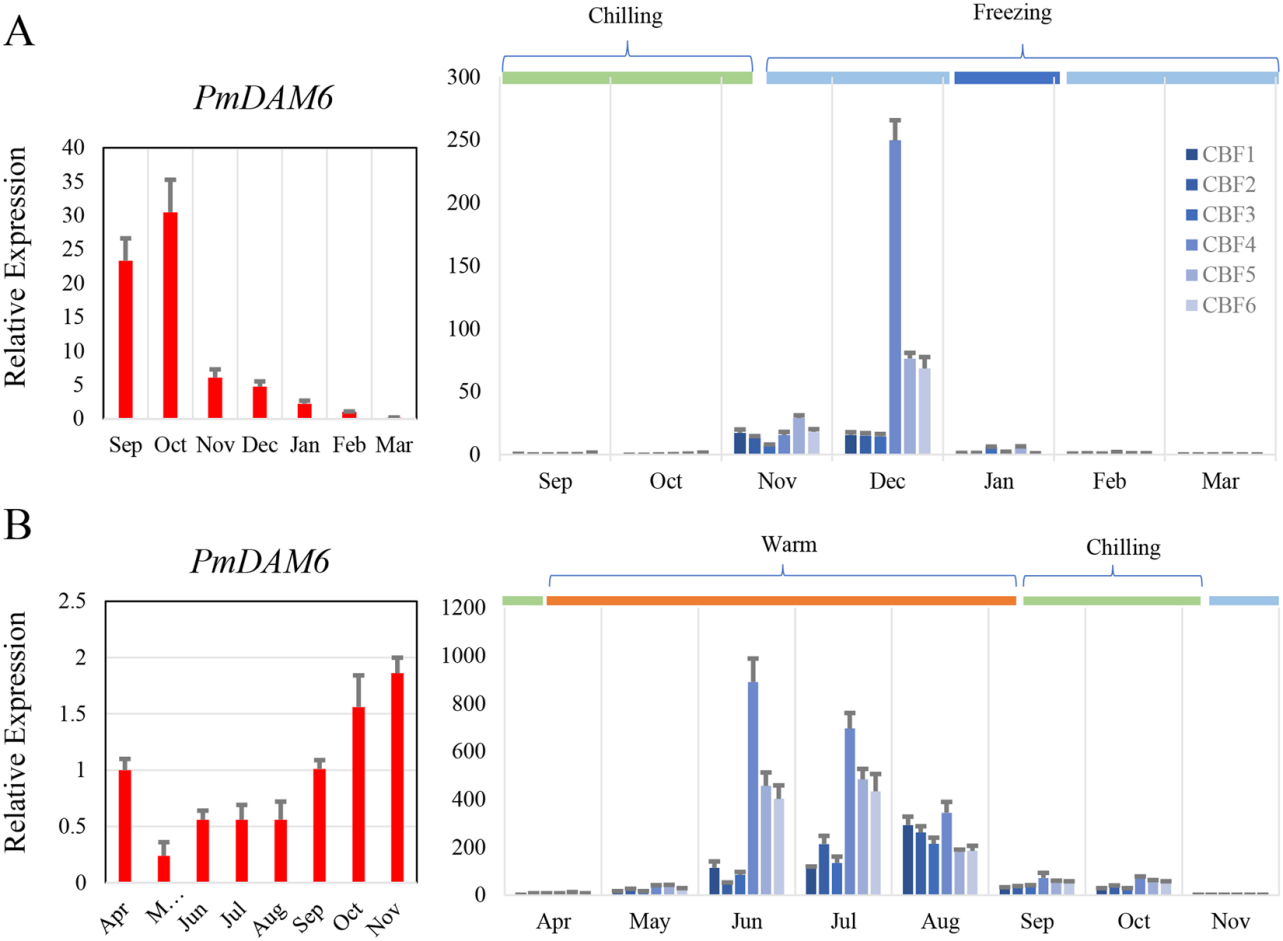
Expression patterns of *PmDAMs* and *PmCBFs* in leaf buds and mature leaves. (**A**) Gene expressions in the leaf buds. (**B**) Gene expressions in the leaves. (Warm: higher than 20 °C, Chilling: lower than 20 °C, Freezing: lower than 0 °C).

The leaf buds experienced a different development process compared to flower bud. They formed in the same period with flower bud, but only enlarged after the flower blooming after dormancy in the cold winter. *DAM* genes were specifically expressed in leaf buds at different developmental stages. *PmDAM1-3* exhibited high expression levels in September, and expression levels of *PmDAM*4*-6* were the highest in October (Fig. 5A). Furthermore, the expression trends of *PmDAMs* in leaf bud were largely consistent at different development stages and showed significant expressions from September to October which then gradually declined from October to March in the next year. However, the expression levels of *PmCBFs* were the highest in November and December. Above all, the expression patterns of *DAM* and *CBF ge*nes between leaf bud and flower bud were largely consistent at different development stages.

The expressions of *PmCBFs* manifested two types of similar patterns with high levels. The first group (*PmCBF1-*3) showed peak expressions in August, up-regulated from April to August, and down-regulated from September to November (Fig. 5B). The second group (*PmCBF4-6*) showed peak expressions in June and July, then declined till November. But all the *PmCBFs* own high levels from June to August.

### Simulated bud dormancy and dormancy release

In order to study the functions of *PmCBF*s and *PmDAMs* in the bud dormancy, we detected the expressions of all six *PmCBF*s and six *PmDAM*s in the simulated condition of bud dormancy and dormancy release.

One year old branches with flower buds were sampled on October 1, 2015. The growing branches were treated with 6 °C ± 2 °C to induce dormancy. The expressions of *PmCBFs* were shown in Fig. 3C, and were of two types. One category of *PmCBFs*, including *PmCBF2*-*5*, showed rising expressions, while the other category, containing*PmCBF1* and *PmCBF6*, showed higher expressions in 10-40 days as compared to 0 day, suggesting the low temperature significantly affect the expressions of *PmCBF*s. However, the expression of *PmDAM6* increased in 0–20 days but dropped in the following days. On first day of low temperature exposure, *PmDAM6* presented a high expression. During the process of dormancy, *PmCBF*s accumulated in low temperature, while the expression of *PmDAM6* declined.

Warm temperature of spring was simulated at 12 °C ± 2 °C to release the bud dormancy with one year old branches on December 10, 2015. After warm temperature treatment, flower buds turned to expand within 10days, and after 20 days, 95% flower buds showed white petals. As shown in Fig. 4B, the expressions of *PmCBFs* and *PmDAM6* reduced overall. The expressions of *PmCBF1*, *PmCBF3*, *PmCBF4* and *PmCBF5* keep stable, but declined remarkablely during 30–40 days. In contrast, *PmCBF2*, *PmCBF6* and *PmDAM6* rapidly declined in 10 days. All results suggested that in the release of dormancy, *PmCBFs* and *PmDAM6* showed a down regulation.

## Discussion

### *PmCBFs* suppress overexpression of *PmDAM6* in flower bud dormancy

Acquirement of protective strategies for reproductive organs in plants possesses vital importance for the continuation of life, however, the uncertain environment changes, like low-temperature stress, remains the major hurdle to the success of plants inhabiting the cold regions. Regulatory pathways and transcriptional factors driving these routings are central to the studies about the success of these plants in fluctuating climatic conditions. *DAM* genes have been shown to be involved in the formation of terminal buds and also intervene growth dynamics in perennial plants like kiwifruit (*Actinidia spp*.), leafy spurge (*Euphorbia esula*), raspberry (*Rubusidaeus*), *Populustrichocarpa*, and peach^17,26–32^. In peach, *PpDAMs* has an association with seasonal dormancy transitions in flower buds^26^. *SVP* genes, orthologs of *DAM*, in *Arabidopsis* show regulatory effect during floral transitions and play a significant role in specifying the floral meristems^33^. In the flower bud of *Prunus mume*, *PmDAM6* exhibited a special expression leading to the dormancy under decreasing temperature (Fig. 3A). Moreover, the appearance of high CBF expression levels enhanced the tolerance of coldness in freezing climates and inhibited the expression of *PmDAM6*. This may lead to a different phenomenon that flowers buds are easier to sense the changes in environment and devise the mechanism of self-protection^34,35^. Nevertheless, this happens only if plants are shocked by cold. When plants were released from cold weathers, *PmDAM6* and *PmCBFs* just relaxed with all decreasing trends and relatively lower levels (Fig. 4). This must be an integrated result from regulation beyond CBFs and DAMs.

In *P*. *persica*, DAMs have shown in vegetative and non-vegetative plant parts development including bud^12^. Here, the protection mechanism prefers to appear in young tissues facing coldness. Annually sampled leaf buds gave important clues about potent roles of *DAM6* and *CBF* genes in *P*. *mume* wherein *PmDAM6* showed high expressions in September to October and the CBFs appeared from November to December (Fig. 5A). Besides, *MdCBF1*, *2* and *4* were discerned in the leaf tissues of apple subjected to cold conditions, and *VrCBF* exhibited a short expression (1/2 h-2 days) both in young and old leaves, while *VrCBF1*, *2* and *3* expressed only in younger tissues^36^. Some reports have indicated a reverse relation between *DAM* and *CBF* during growth curves, and dormancy control, wherein *FT* can be down-regulated by the high expression of *DAM*^30^. This may provide an explanation for the growth and dormancy of young tissues.

In the mature leaf aging process, *PmDAM6* also showed a negative correlation with *PmCBFs* especially for *PmCBF4-6*, though this happened under a warm environment. However, it is confirmed that CBFs function in heat and drought as well as cold response in soybean^37^. The ectopically expressing *PpCBF1* induced aging in leaf and also delayed bud opening in the spring^24^.

Under artificial control environment, *PpDAM5* and *PpDAM6* exhibited an upregulation under sustained low temperature and showed a downregulation by warm temperature in peach^38^. The growth of peach buds are negatively correlated with the expression of *PpDAM5* and *PpDAM6*^13,39^. In addition, the sustained low temperature could satisfy the needs of a chilling requirement in buds to promote the release of dormancy with *PpDAM5* and *PpDAM6* down regulated^16^. In *P*. *mume*, high expression of *PmDAM6* hastened the flower buds into dormancy. With the accumulation of coldness in winter, the expressions of *PmCBF*s increased, and that of *PmDAM6* decreased, indicating that high amount of *PmCBF*s seemed to depress the expression of *PmDAM6*. When warm temperature spell in spring, the flower bud successfully released from cold dormancy with both *PmCBFs* and *PmDAM6* declined. All these observations push us to establish the opposite regulations from PmCBFs to PmDAM6

### Interactions among PmDAMs and PmCBFs

**Figure 6:**
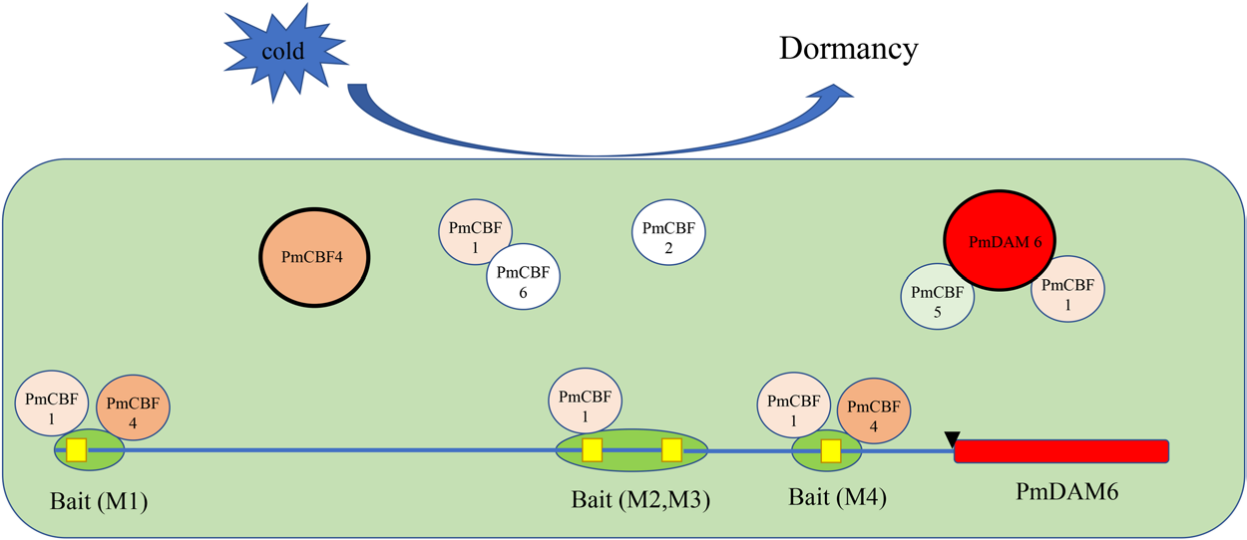
Molecular regulation model of PmDAMs and PmCBFs during flower bud dormancy. PmCBF4 and PmDAM6 could form homodimers, respectively (displayed in larger circles). PmCBF5 could form heteromeric complexes with PmDAM1 and PmDAM6. Meanwhile, PmCBF1 and PmCBF3 recognized the promoter of PmDAM6 by the binding site M2 and M3, PmCBF1 and PmCBF4 could discern M1 and M4.

As reported in recent researches, CBF could bind to the promoter of *DAM* genes based on a fixed motif. For the model plant, *Arabidopsis*, CBFs recognize DNA sequences with CRT/DRE sites, which cover a core motif of CCGAC. Compared with other herbaceous plant, like oilseed rape, wheat, or rye, the cold response pathway with a member of CBF remains conservative between species suggesting its vital place for the whole pathway. In pear, the interaction between PpCBF and the promoter of *PpDAMs* give a convincing proof. In *Pyrus pyrifolia*, the CRT/DRE binding ability between PpCBF and the promoters of *PpDAM1*and *PpDAM3* was verified^6^. In the genome of *P*. *mume*, the conserved binding motif exists in the promoters of *PmDAM1*, *PmDAM4*, *PmDAM5*,and *PmDAM6*. Particularly, it makes the function of *PmDAMs* more complex that the *PmDAM6* promoter contain more CRT/DRE core sites (Fig. 1). For pear, PpCBF does not bind to the bait, when CGGC was transformed into AAAA, suggesting the regulation of *DAM* depends on the recognition of this core sequence. In our study, Y1H assays exhibited PmCBF1, PmCBF3 and PmCBF4 indeed interacted with the promoters of *PmDAM6* by different sites. But PmCBF3 was not only located in the nucleus but also the chloroplast. Besides, PmCBF5 could form heteromeric complexes with PmDAM1 as well as PmDAM6, suggesting that PmDAMs and PmCBFs might function in the bud dormancy by dimerizations (Fig. 6). Therefore, scrutinizing the expression patterns coupled with dimerizations trends can be a reasonable platform to grasp detailed understanding of protein-protein interactions among PmDAMs and PmCBFs during bud transitions. Though the PmCBFs may have matching functions^20^, the alternative interaction abilities still provided a possible regulation from chaos. Especially for the *PmDAM6*, the expression did not get the bottom, when the *PmCBFs* exaggerated. Conversely, the expression of *PmDAM6* kept a relative stabilization, which drove us to hypothesize that the interaction from protein to protein and protein to DNA conducted a feedback loop in the deep dormancy condition (Fig. 6). However, there were still mysteries because of the multiple CRT/DRE core sites that whether the number of binding sites superpose or subduct the function of downstream gene, all the hypotheses need further researches.

In our study, we observed the crossover of the expression between PmCBFs and PmDAM6, and provided the evidences that besides multiple recognitions to the promoter of PmDAM6, PmCBFs also could form protein complex with PmDAM6, which act as a center role in dormancy induction. This multiplex regulation might reflect the evolvement of plant towards more rapidly changing environment. Moreover, the expression patterns in vegetative bud and simulated dormancy test of bud convince the negative control in the dormancy formation. We believe this novel research will help direct future discussion on the interactive role of *DAM* and *CBF* genes as essential regulators of bud dormancy.

## Materials and Methods

### Plant Material

*P*. *mume* ‘Sanlun Yudie’, an early flowering cultivar, was selected as plant material from the Beijing Forestry University, in Beijing, China (40° 00′ N, 116° 18′ E). These samples were taken from flower bud (from July, 2015 to February, 2016; collected a sample every 30 days; Supplementary Fig. S1), flower (early flowers in three different morphologies, full blooming, sepal, petal, stamen, and pistil, in March, 2016; Supplementary Fig. S2), leaf bud (from September, 2015 to March, 2016; collected a sample every 30 days), leaf (from April, 2016 to November, 2016; collected a sample every 30 days). For the dormancy tests (8 °C for 10 hours during daytime and 4 °C for 14 hours at night) and dormancy releasing tests (14 °C for 10 hours during daytime and 10 °C for 14 hours at night), thirty branches were cut to simulate the tests on October 1, 2015 and December 10, 2015, respectively. In these simulation tests, the flower buds were sampled every 10 days. All samples were immediately immersed in liquid nitrogen and were stored at −80 °C for RNA extraction. Total RNA was extracted by TRIzol reagent (Aidlab, China).

### Real-time quantitative PCR

Full length cDNA sequences of six *PmDAMs* and six *PmCBFs* (Supplementary Data S1) were amplified through PCR using the primers in Supplementary Table S1. PikoReal real-time PCR system (Thermo Fisher Scientific, Germany) was used to investigate the expressions of *PmDAMs* and *PmCBFs* in different organs. The primers of RT-qPCR experiments were shown in Supplementary Table S2 and the experiments were carried out using previous method^40^. *PmPP2A* (*protein phosphatase 2A*) was considered as reference gene^41^. Three biological replicates were performed to calculate the standard deviation. The correlations between gene expressions were done by spearman method, and significant was analysed with kruskal-wallis test in R.

### Gene cloning and Yeast 2 Hybrid assays

Full length cDNA sequences of *PmDAMs* and *PmCBFs* were amplified through PCR using specific primers (Supplementary Table S3). These sequences were cloned into pGBKT7 bait vector and pGADT7 prey vector at the EcoRI and BamHI sites, respectively, using an In-Fusion HD Cloning Kit System (Clonetech, USA). The Y2H assays were performed by former methods^42^. Each interaction analysis was performed three times.

### BiFC Assays

Specific primers were used for BiFC assessment (Supplementary Table S4). Full length cDNA sequences of *PmDAMs* and *PmCBFs* were cloned, pair-wise, into pCambia1300-YFP-C and pCambia1300-YFP-N to get BiFC constructs. Coexpression analysis was carried out on the leaves of *Nicotiana benthamiana* according to the procedure stated by Schutze *et al*.^43^. Chimeric fluorescence, emitted by fusion proteins, was examined under Leica TCS SP8 Confocal Laser Scanning Platform with YFPs being motivated at 514 nm.

### Promoter cloning and Yeast 1 Hybrid assays

The genomic DNA of *P*. *mume* was isolated from flower bud using DNAsecure Plant Kit (DP320-02, TianGen, China). 2 kb up-stream promoter sequence of *PmDAM6*was extracted by PCR using specific primers (Supplementary Table S5) and the plasmid of pMDTM18-T-proDAM6 was obtained by former method^42^. In 2 kb promoter of *PmDAM6*, there are four CCGAC sequences called CBF binding site. According these four CBF binding sites, six baits were designed. Three of them were obtained from the original genome, then each of them was duplicated to form the follow three baits. All six fragments were imported to the pAbAi-bait vectors. The special primers related to cloning these fragments were shown in Supplementary Table S6. The plasmids of pGADT7-*PmCBF*s were obtained in Y2H assays. These plasmids were transformed into the Y1H Gold strains containing pAbAi-bait, respectively, and these tests were screened on SD/-Leu/AbA plates. All transformations and screenings were observed in triplicate. These Y1H assays were executed by a Matchmaker Gold Yeast One-Hybrid System kit (Clontech, America) following its user manual and correlation steps.

## Electronic supplementary material

Supplementary Dataset 1
