# Supplementary material for: PmCBFs synthetically affect PmDAM6 by alternative promoter binding and protein complexes towards the dormancy of bud for Prunus mume
Source: Sci Rep. 2018 Mar 14;8:4527. doi: 10.1038/s41598-018-22537-w (PMC5852209; doi:10.1038/s41598-018-22537-w)
Supplement: Supplementary file 1 — Supplementary Dataset 1 [file 41598_2018_22537_MOESM1_ESM.docx]

Supplementary Information

***PmCBFs* synthetically affect *PmDAM6* by alternative promoter binding and protein complexes towards the dormancy of bud for *Prunus mume***

Kai Zhao, Yuzhen Zhou, Sagheer Ahmad, Xue Yong, Xuehua Xie, Yu Han, Yushu Li, Lidan Sun, Qixiang Zhang*

**Supplementary Information**

Supplementary Fig. S1. The flower bud of *P. mume* ‘Sanlun Yudie’ in July, August, September, October, November, December, January, and February.

Supplementary Fig. S2. The bloom process and different flower structures of *P. mume* ‘Sanlun Yudie’.

Supplementary Fig. S3. Control experiments of BiFC assays.

Supplementary Fig. S4. Testing of bait vectors for autoactivation.

Supplementary Table S1. Primers used for cloning.

Supplementary Table S2. Primer used for real-time quantitative RT-PCR.

Supplementary Table S3. Primer used in PCR reactions for Yeast two-hybrid assays.

Supplementary Table S4. Primer used in PCR reactions for BiFC assays.

Supplementary Table S5. Primer used in PCR reactions for the cloning of the 2kb promoter sequence of *PmDAM6*.

Supplementary Table S6. Primer used in PCR reactions for yeast one-hybrid assays.

Supplementary Data S1. The cDNA sequences of six *PmCBFs* and six *PmDAMs* cloned from *P. mume* ‘Sanlun Yudie’.

Supplementary Data S2. The 2kb up-stream promoter sequence of *PmDAM6*.

Supplementary Data S3. The sequences of pAbAi-genes.

# Supplementary Figures and Tables

## Supplementary Figures

**Supplementary Fig. S1.** The flower bud of *P. mume* ‘Sanlun Yudie’ in July, August, September, October, November, December, January, and February.


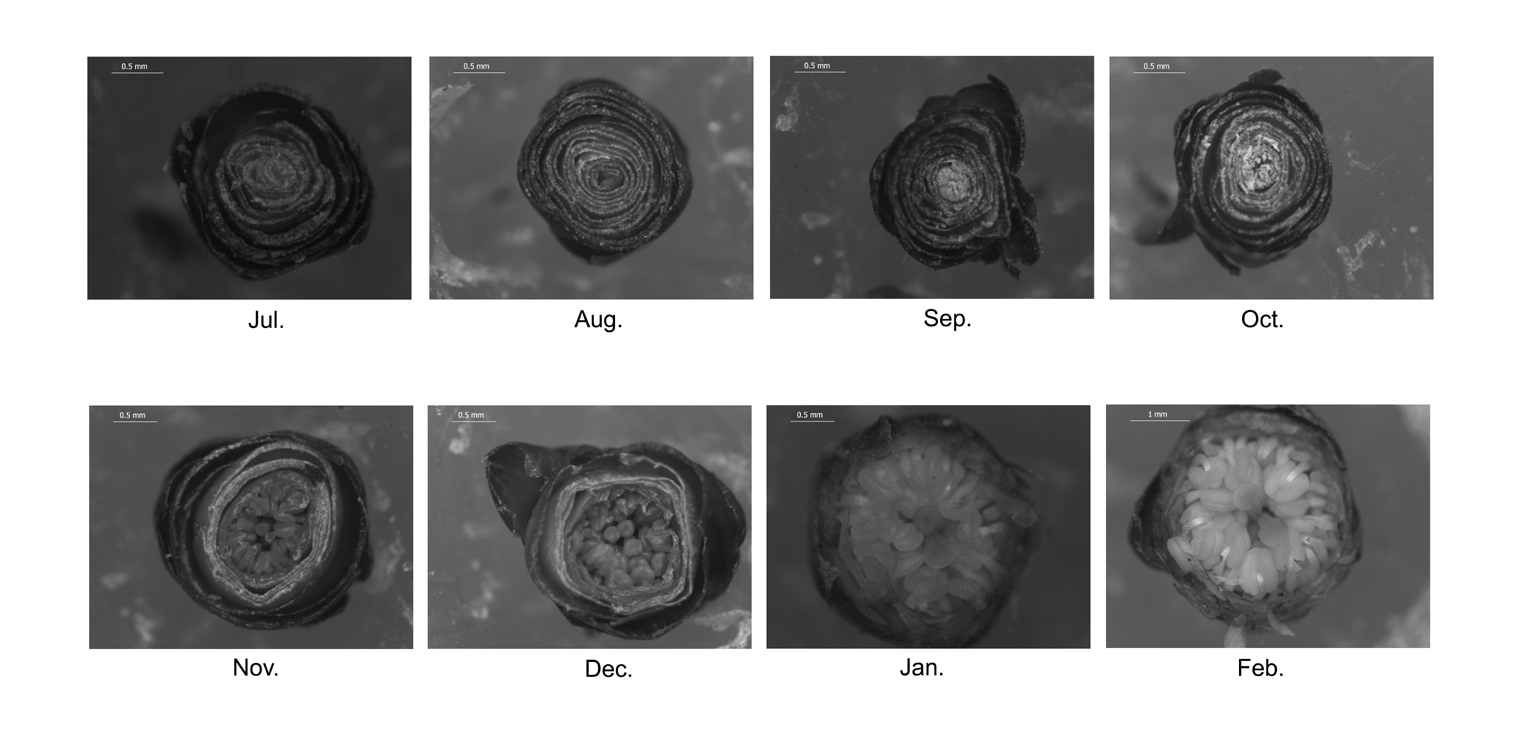


**Supplementary Fig. S2.** The bloom process and different flower structures of *P. mume* ‘Sanlun Yudie’. F1: Small flower bud; F2: Big flower bud; F3: First blooming; F4: Full blooming; Se: Sepal; Pe: Petal; St: Stamen; Ca: Carpel.


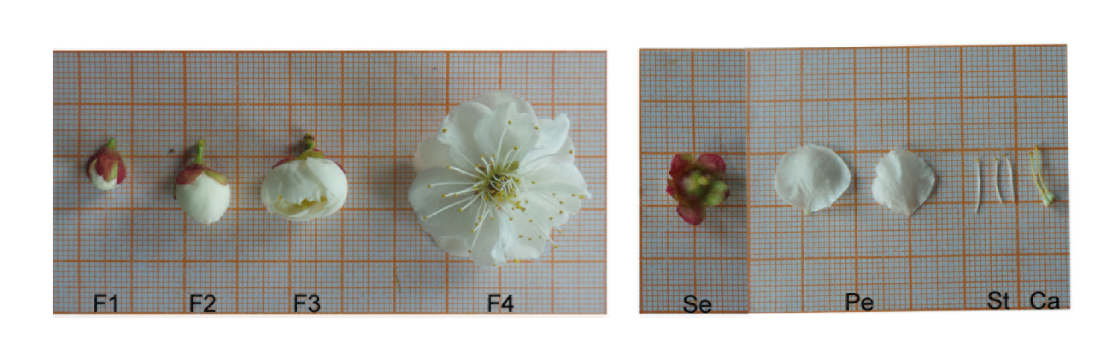


**Supplementary Fig. S3.** **Control experiments of BiFC assays.** In every interactions, the two proteins were fused with either the C or N terminus of yellow fluorescent protein (YFP; designated as YFP^C^ or YFP^N^, respectively). Different combinations of the fused constructs were co-transformed into leaf cell of *N. benthamiana*, and then the cells were observed by confocal microscopy. There was no interactions in PmDAM1-YFP^N^/YFP^C^, YFP^C^/PmCBF5-YFP^N^, PmCBF5-YFP^C^/YFP^N^, and YFP^N^/ PmDAM6-YFP^C^. Bright field and YFP were excited at 514 nm. The red fluorescent showed the chloroplast position.


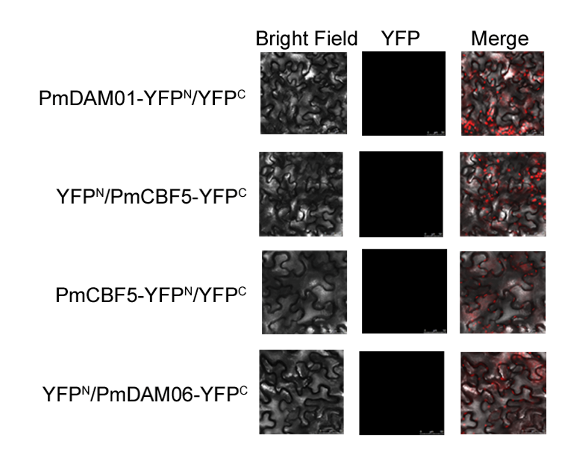


**Supplementary Fig. S4.** **Testing of bait vectors for autoactivation.** There were six baits in Yeast one-hybrid assays, including pAbAi-1-1, pAbAi-1-3, pAbAi-2-1, pAbAi-2-3, pAbAi-3-1, and pAbAi-3-3.


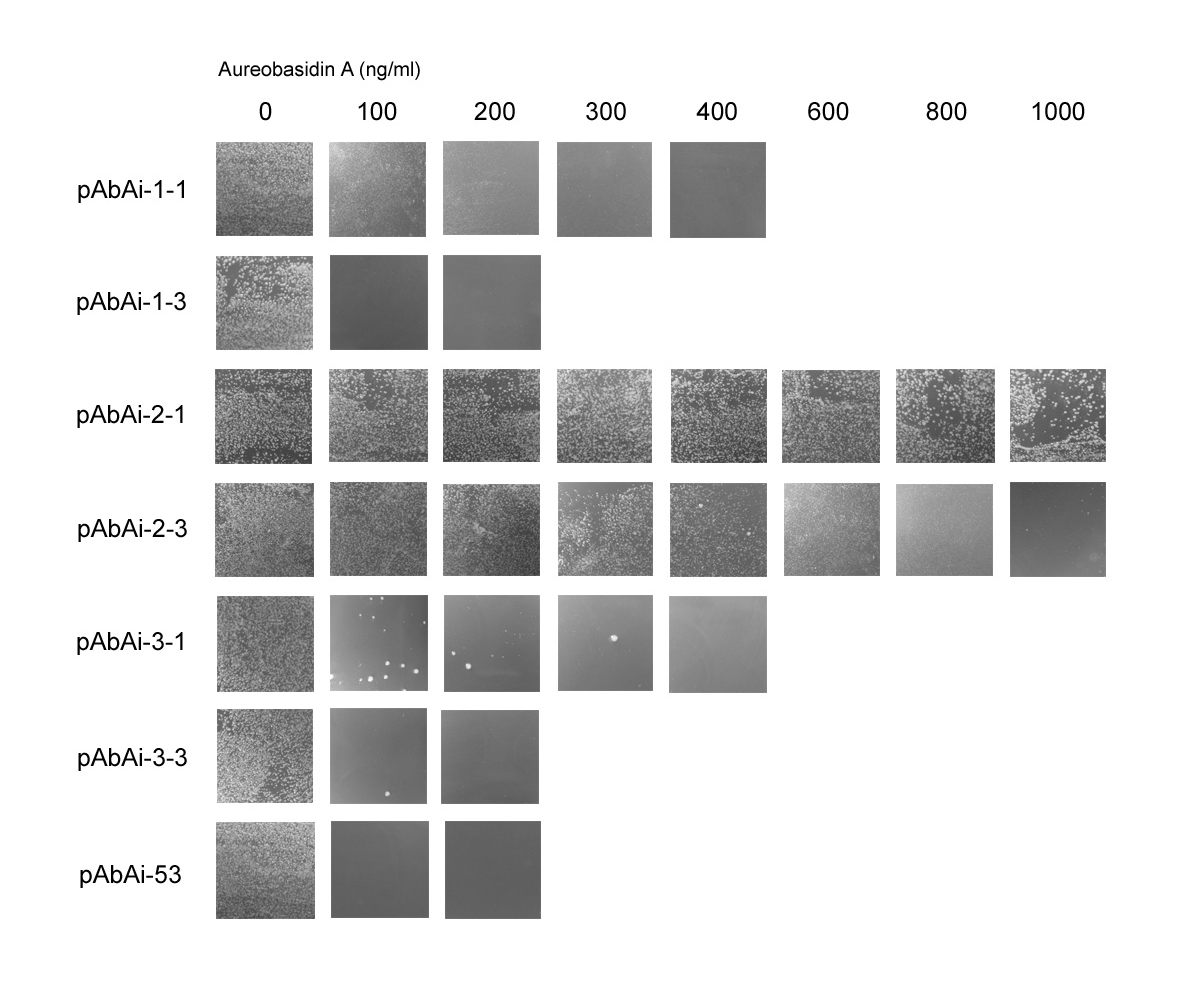


## Supplementary Tables

**Supplementary Table S1.** Primers used for cloning.

| Gene | Forward primer | Reverse primer | Annealing temperature | |
| --- | --- | --- | --- | --- |
| *PmCBF1* | 5' ATGCACAGGTTCTTGTCTGAT 3' | 5' TTAATTGGAGAAACTCCACAA 3' | | 56 ℃ |
| *PmCBF2* | 5' ATGGATATGATCTACAGCCAG 3' | 5' TCAAATAGAAAAACTCCACAG 3' | | 53 ℃ |
| *PmCBF3* | 5' ATGGCTGCTCGTGCCCATGAC 3' | 5' TTAAATGGAGAAATTCCACAATTTGA 3' | | 64 ℃ |
| *PmCBF4* | 5' ATGGACGTGTCTGAACTTTCC 3' | 5' TTAAATGGAGAAACTCCACAA 3' | | 57 ℃ |
| *PmCBF5* | 5' ATGGACACGATCTTCTCTCAG 3' | 5' TCAGATAGAGAAACTCCACAA 3' | | 56 ℃ |
| *PmCBF6* | 5' ATGGCTGCTCGTGCCCATGAC 3' | 5' TCAGATAGAGAAACTCCACAA 3' | | 64 ℃ |
| *PmDAM1* | 5'ATGAAAATGATGAGGGAGAAG3' | 5' TTATGGAAGCCCCAGTTTGAG3' | | 52 ℃ |
| *PmDAM2* | 5'ATGGTGAAGACGATGAGGAAG3' | 5' TTAGGGAAGCCCCAGTTTGAG3' | | 56 ℃ |
| *PmDAM3* | 5'ATGATGAGGAAGAAGATCAAG3' | 5' TTAAGGAAGCCCCAGTTCGAG3' | | 51 ℃ |
| *PmDAM4* | 5'ATGGTGAAAATGATGAGGGAG3' | 5' TTAGGAACGCCCCAGTTTGAG3' | | 54 ℃ |
| *PmDAM5* | 5'ATGATGAATAAGATCAAGATC3' | 5' TTAACGCCCCAGTTTGAGAGA3' | | 47 ℃ |
| *PmDAM6* | 5'ATGGTGAAAATGATGAGGGAG3' | 5' CTAGGGAAGCCCCAGTTTGAG3' | | 59 ℃ |

**Supplementary Table S2.** Primer used for real-time quantitative RT-PCR.

| Gene | Forward primer | Reverse primer |
| --- | --- | --- |
| *PmPP2A* | 5'AGGGTTCGGCTCGCAATAATAGA3' | 5'TGTTAGCAGCAGCATCACGAAT3' |
| *PmCBF1* | 5' ATGCACAGGTTCTTGTCTGAT 3' | 5' CGTCGGACCAAGAAGTCCTTA 3' |
| *PmCBF2* | 5' AAAACCCGGATAAGTCGTCGT 3' | 5' ATGTCCTGGCCTTCTTGTTGT 3' |
| *PmCBF3* | 5' TGCATCAATTTCGCTGATTCC 3' | 5' CGCTGCACACTCCACCAAATT 3' |
| *PmCBF4* | 5' ACGTGTCTGAACTTTCCGACT 3' | 5' GCTTGACGCCAATATGACTTC 3' |
| *PmCBF5* | 5' TTCTCTCAGCTTTCTGACTCG 3' | 5' AACCGGATGCCTCGTCTCTTT 3' |
| *PmCBF6* | 5' TCCGGTTGGAGGCTGCCGGTG 3' | 5' TTCCCATATCTACAATAGCCT 3' |

| *PmDAM1* | 5'AGTATGAAGGATGTTATTCAA3' | 5'CTTAAGTTCCTTGCTCAATCT3' |
| --- | --- | --- |
| *PmDAM2* | 5'AACCAGCTACGGCAGAGGATG3' | 5'AGATTCAGATGACATGCCTT3' |
| *PmDAM3* | 5'TCGGATTGAGCAAGGAACTGG3' | 5'CATTCTCAGTTCTTCCTTTGT3' |
| *PmDAM4* | 5'ACCCTTGTCCGTGTGATGGAA3' | 5'ATCACCATCTGATTGTTGCCT3' |
| *PmDAM5* | 5'AGGCTGAATAATAATATTGAA3' | 5'TTAACGCCCCAGTTTGAGAGA3' |
| *PmDAM6* | 5'AACCAACAACCAGTTAAGGCATA3' | 5'CAATTACGGCAGATTCAGATGA3' |

**Supplementary Table S3.** Primer used in PCR reactions for yeast two-hybrid assays.

| **Gene** | **Sequence 5’-3’** | **vector** |
| --- | --- | --- |
| BK-PmCBF2-F | CATGGAGGCCGAATTCATGGATATGATCTACAGCCAG | pGBKT7 |
| BK-PmCBF2-R | GCAGGTCGACGGATCCTCAAATAGAAAAACTCCACAG |  |
| BK-PmCBF3-F | CATGGAGGCCGAATTCATGGCTGCTCGTGCCCATGAC | pGBKT7 |
| BK-PmCBF3-R | GCAGGTCGACGGATCCTTAAATGGAGAAATTCCACAA |  |
| BK-PmCBF4-F | CATGGAGGCCGAATTCATGGACGTGTCTGAACTTTCC | pGBKT7 |
| BK-PmCBF4-R | GCAGGTCGACGGATCCTTAAATGGAGAAACTCCACAA |  |
| BK-PmCBF5-F | CATGGAGGCCGAATTCATGGACACGATCTTCTCTCAG | pGBKT7 |
| BK-PmCBF5-R | GCAGGTCGACGGATCCTCAGATAGAGAAACTCCACAA |  |
| BK-PmCBF6-F | CATGGAGGCCGAATTCATGGCTGCTCGTGCCCATGAC | pGBKT7 |
| BK-PmCBF6-R | GCAGGTCGACGGATCCTCAGATAGAGAAACTCCACAA |  |
| AD-PmCBF2-F | GGAGGCCAGTGAATTCATGGATATGATCTACAGCCAG | pGADT7 |
| AD-PmCBF2-R | CGAGCTCGATGGATCCTCAAATAGAAAAACTCCACAG |  |
| AD-PmCBF3-F | GGAGGCCAGTGAATTCATGGCTGCTCGTGCCCATGAC | pGADT7 |
| AD-PmCBF3-R | CGAGCTCGATGGATCCTTAAATGGAGAAATTCCACAA |  |
| AD-PmCBF4-F | GGAGGCCAGTGAATTCATGGACGTGTCTGAACTTTCC | pGADT7 |
| AD-PmCBF4-R | CGAGCTCGATGGATCCTTAAATGGAGAAACTCCACAA |  |
| AD-PmCBF5-F | GGAGGCCAGTGAATTCATGGACACGATCTTCTCTCAG | pGADT7 |
| AD-PmCBF5-R | CGAGCTCGATGGATCCTCAGATAGAGAAACTCCACAA |  |
| AD-PmCBF6-F | GGAGGCCAGTGAATTCATGGCTGCTCGTGCCCATGAC | pGADT7 |
| AD-PmCBF6-R | CGAGCTCGATGGATCCTCAGATAGAGAAACTCCACAA |  |
| BK-PmDAM1-F | CATGGAGGCCGAATTCATGAAAATGATGAGGGAGAAG | pGBKT7 |
| BK-PmDAM1-R | GCAGGTCGACGGATCCTTATGGAAGCCCCAGTTTGAG |  |
| BK-PmDAM2-F | CATGGAGGCCGAATTCATGGTGAAGACGATGAGGAAG | pGBKT7 |
| BK-PmDAM2-R | GCAGGTCGACGGATCCTTAGGGAAGCCCCAGTTTGAG |  |
| BK-PmDAM3-F | CATGGAGGCCGAATTCATGATGAGGAAGAAGATCAAG | pGBKT7 |
| BK-PmDAM3-R | GCAGGTCGACGGATCCTTAAGGAAGCCCCAGTTCGAG |  |
| BK-PmDAM4-F | CATGGAGGCCGAATTCATGGTGAAAATGATGAGGGAG | pGBKT7 |
| BK-PmDAM4-R | GCAGGTCGACGGATCCTTAGGAACGCCCCAGTTTGAG |  |
| BK-PmDAM5-F | CATGGAGGCCGAATTCATGATGAATAAGATCAAGATC | pGBKT7 |
| BK-PmDAM5-R | GCAGGTCGACGGATCCTTAACGCCCCAGTTTGAGAGA |  |
| BK-PmDAM6-F | CATGGAGGCCGAATTCATGGTGAAAATGATGAGGGAG | pGBKT7 |
| BK-PmDAM6-R | GCAGGTCGACGGATCCCTAGGGAAGCCCCAGTTTGAG |  |
| AD-PmDAM1-F | GGAGGCCAGTGAATTCATGAAAATGATGAGGGAGAAG | pGADT7 |
| AD-PmDAM1-R | CGAGCTCGATGGATCCTTATGGAAGCCCCAGTTTGAG |  |
| AD-PmDAM2-F | GGAGGCCAGTGAATTCATGGTGAAGACGATGAGGAAG | pGADT7 |
| AD-PmDAM2-R | CGAGCTCGATGGATCCTTAGGGAAGCCCCAGTTTGAG |  |
| AD-PmDAM3-F | GGAGGCCAGTGAATTCATGATGAGGAAGAAGATCAAG | pGADT7 |
| AD-PmDAM3-R | CGAGCTCGATGGATCCTTAAGGAAGCCCCAGTTCGAG |  |
| AD-PmDAM4-F | GGAGGCCAGTGAATTCATGGTGAAAATGATGAGGGAG | pGADT7 |
| AD-PmDAM4-R | CGAGCTCGATGGATCCTTAGGAACGCCCCAGTTTGAG |  |
| AD-PmDAM5-F | GGAGGCCAGTGAATTCATGATGAATAAGATCAAGATC | pGADT7 |
| AD-PmDAM5-R | CGAGCTCGATGGATCCTTAACGCCCCAGTTTGAGAGA |  |
| AD-PmDAM6-F | GGAGGCCAGTGAATTCATGGTGAAAATGATGAGGGAG | pGADT7 |
| AD-PmDAM6-R | CGAGCTCGATGGATCCCTAGGGAAGCCCCAGTTTGAG |  |

**Supplementary Table S4.** Primer used in PCR reactions for BiFC assays.

| **Gene** | **Sequence 5’-3’** |
| --- | --- |
| BiFC-PmCBF2-F | TGCAGGGAGGAGGATCCATGGATATGATCTACAGCCAG |
| BiFC-PmCBF2-R | CGGTGCACTAGTGTCGACAATAGAAAAACTCCACAG |
| BiFC-PmCBF3-F | TGCAGGGAGGAGGATCCATGGCTGCTCGTGCCCATGAC |
| BiFC-PmCBF3-R | CGGTGCACTAGTGTCGACAATGGAGAAATTCCACAA |
| BiFC-PmCBF4-F | TGCAGGGAGGAGGATCCATGGACGTGTCTGAACTTTCC |
| BiFC-PmCBF4-R | CGGTGCACTAGTGTCGACAATGGAGAAACTCCACAA |
| BiFC-PmCBF5-F | TGCAGGGAGGAGGATCCATGGACACGATCTTCTCTCAG |
| BiFC-PmCBF5-R | CGGTGCACTAGTGTCGACGATAGAGAAACTCCACAA |
| BiFC-PmCBF6-F | TGCAGGGAGGAGGATCCATGGCTGCTCGTGCCCATGAC |
| BiFC-PmCBF6-R | CGGTGCACTAGTGTCGACGATAGAGAAACTCCACAA |
| BiFC-PmDAM1-F | TGCAGGGAGGAGGATCCATGAAAATGATGAGGGAGAAG |
| BiFC-PmDAM1-R | CGGTGCACTAGTGTCGACTGGAAGCCCCAGTTTGAG |
| BiFC-PmDAM2-F | TGCAGGGAGGAGGATCCATGGTGAAGACGATGAGGAAG |
| BiFC-PmDAM2-R | CGGTGCACTAGTGTCGACGGGAAGCCCCAGTTTGAG |
| BiFC-PmDAM3-F | TGCAGGGAGGAGGATCCATGATGAGGAAGAAGATCAAG |
| BiFC-PmDAM3-R | CGGTGCACTAGTGTCGACAGGAAGCCCCAGTTCGAG |
| BiFC-PmDAM4-F | TGCAGGGAGGAGGATCCATGGTGAAAATGATGAGGGAG |
| BiFC-PmDAM4-R | CGGTGCACTAGTGTCGACGGAACGCCCCAGTTTGAG |
| BiFC-PmDAM5-F | TGCAGGGAGGAGGATCCATGATGAATAAGATCAAGATC |
| BiFC-PmDAM5-R | CGGTGCACTAGTGTCGACACGCCCCAGTTTGAGAGA |
| BiFC-PmDAM6-F | TGCAGGGAGGAGGATCCATGGTGAAAATGATGAGGGAG |
| BiFC-PmDAM6-R | CGGTGCACTAGTGTCGACGGGAAGCCCCAGTTTGAG |

**Supplementary Table S5.** Primer used in PCR reactions for the cloning of the 2kb promoter sequence of *PmDAM6*.

| **Name** | **Sequence 5’-3’** |
| --- | --- |
| ProDAM6-F | AAAAGAAAAACTACGAATTGC |
| ProDAM6-R | TTCCCTTCAAAAAAATTCAAA |

**Supplementary Table S6.** Primer used in PCR reactions for yeast one-hybrid assays. The sequence of Pro1-1 were cloned by the primers including BK-Pro1-1-F, BK-Pro1-1-R, BK-Pro1-2-F, and BK-Pro1-2-R. The sequence of Pro1-3 were cloned by the primers including BK-Pro1-1-F and BK-Pro1-2-R. The sequence of Pro2-1 were cloned by the primers including BK-Pro2-1-F, BK-Pro2-1-R, BK-Pro2-2-F, and BK-Pro2-2-R. The sequence of Pro2-3 were cloned by the primers including BK-Pro2-1-F and BK-Pro2-2-R. The sequence of Pro3-1 were cloned by the primers including BK-Pro3-1-F, BK-Pro3-1-R, BK-Pro3-2-F, and BK-Pro3-2-R. The sequence of Pro3-3 were cloned by the primers including BK-Pro3-1-F and BK-Pro3-2-R.

| **Name** | **Sequence 5’-3’** | **Utilization** |
| --- | --- | --- |
| BK-Pro1-1-F | AAGCTTGAATTCGAGCTCACCTGTCTCCATCCGACT | Cloning the sequences of Pro1-1 and Pro1-3 into pAbAi bait vector. |
| BK-Pro1-1-R | AGTCGGATGGAGACAGGTTTTAGTTTGTTGTCGGTT |  |
| BK-Pro1-2-F | AACCGACAACAAACTAAAACCTGTCTCCATCCGACT |  |
| BK-Pro1-2-R | CATGCCTCGAGGTCGACTTTAGTTTGTTGTCGGTT |  |
| BK-Pro2-1-F | AAGCTTGAATTCGAGCTCAATTCTCTTAGTTTTTCT | Cloning the sequences of Pro2-1 and Pro2-3 into pAbAi bait vector. |
| BK-Pro2-1-R | AGAAAAACTAAGAGAATTAATGTTTACCTTTGTCGG |  |
| BK-Pro2-2-F | CCGACAAAGGTAAACATTAATTCTCTTAGTTTTTCT |  |
| BK-Pro2-2-R | CATGCCTCGAGGTCGACAATGTTTACCTTTGTCGG |  |
| BK-Pro3-1-F | AAGCTTGAATTCGAGCTCAAAAGAAAAACTACGAAT | Cloning the sequences of Pro3-1 and Pro3-3 into pAbAi bait vector. |
| BK-Pro3-1-R | ATTCGTAGTTTTTCTTTTTTAAAAACATGTCGGTGG |  |
| BK-Pro3-2-F | CCACCGACATGTTTTTAAAAAAGAAAAACTACGAAT |  |
| BK-Pro3-2-R | CATGCCTCGAGGTCGACTTAAAAACATGTCGGTGG |  |

# Supplementary Data

**Supplementary Data S1.** The cDNA sequences of six *PmCBFs* and six *PmDAMs* cloned from *P. mume* ‘Sanlun Yudie’.

> PmCBF1

ATGCACAGGTTCTTGTCTCATTTTTCTGACTCCGTCGACCAGCCCGACTCAAGTTCGTTGTCCGACGCCAGCGTCACGACTCTAAGGACTTCTTGGTCCGACGAGGACGTCATATTGGCGTCGAGCCGACCAAAGAAGCGAGCTGGGAGGAGGGTTTTCAAGGAGACCAGGCACCCTGTTTATAGGGGCGTGAGGAGGAGGAACAATGACAAGTGGGTGTGTGAAATGAGAGAGCCCAAGAAGACGAAGTCCAGGATATGGCTCGGGACTTATCCGACGGCGGAGATGGCTGCTCGTGCACATGACGTGGCGGCATTGGCATTTAGAGGGAAGCTTGCCTGCCTCAACTTCGCTGACTCCGCGTGGAGGCTGCCCGTGCCTGCTTCCATGGATGCAATGGATATTCGGAGAGCGGCCTCCGAGGCAGCTGAGGGGTTTAGGCCGGTGGAGTTTGGTGGAGTGTCCAGCAGCAGCAGTGATGAGAAGGAGAGTATGGTGGTGCAGGTGGAAGAGAAGAAGAAGAAGGGTAGTGTGAATATGGAAAGAAGCAGAAGCTTGAGCTTGTCCTATTGGGATGAGGAGGAAGTGTTGGACATGCCAAGGATGCTTGATCACATGGCTCAAGGCCTTCTTCTTTCTCCACCTCAATGCTTAGGTGGCTACATTTGGGATGACATGGGAACCGATGCTGATGTCGAATTGTGGAGTTTCTCCAATTAA

> PmCBF2

ATGGATATGATCTACAGCCAGGTCTCTGATTTGGCTTCTCTGGAAAACCCGGATAAGTCGTCGTTTTCGGACGCCAGCGTCACGGCCCGGCGAGCTTCTCTTTCAGATGAGGCGGTCATACTGGCGTCCAGCTGCCCGAAAAGGCGGGCGGGGAGGAGGGTTTTCAAGGAGACCAGGCACCCGGTTTATAGGGGTGTGAGGAGGAGGAACAACAACAAGTGGGTGTGTGAGCTGAGAGAGCCCAACAACAAGAAGGCCAGGACATGGCTCGGGACTTATCCGACAGCTGATATGGCGGCTCGTGCCCATGATGTCGCTGCATTGGCGTTTAGGGGGAAGCTTGCCTGCCTCAACTTTGCTGACTCGGCTTGGCCGCTGCCCGTGCCGGCCTCCACCGATGCCGCGGGGATTAGGAGGGCGGCCACCGAGGCGGTTGAAGCGTTTAGGCAGGCGGAGGATGGTGGTGTTTATGAGAAGGAGAGTAAGGCGGTGGTGAGCGAGGAGAAGGGTTGTGTAGAAATGGAGGGAAGCAGCAACTTTTTTTATTTGGACGAGGAGGAAATATTTGAGATGCCAAGGTTGCTTGATGACATGGCTGAAGGGCTTATGCTTTGTCCACCTCAATGTTTAGATGGCCACATGGATTGGAATGACGTGGAAACTGATGATGATTTGAAACTGTGGAGTTTTTCTATTTGA

> PmCBF3

ATGGCTGCTCGTGCCCATGACGTGGCGGCATTGGCGTTTAGAGGGAAGCTTGCCTGCATCAATTTCGCTGATTCCGCGTGGAGGCTGCCTGTGCCGGCTTCCATGGATACCATGGATATTCGGAGAGCTGCCGCGGAAGCAGCTGAGGGGTTTAGGCCGGCGGAGTTTGGTGGATTGTGCAGCGGCAGCAGTGATGAGAAGGAGAGAATGGTGGTGCAGGTGGAAGAGAAAAACAAGAAGGGTAGTGTGAACTTGGAAAGAAGCAGAAGCTTGAGTTTGTCCTATTGGGATGAGGAGGAAGTGTTTGACATGCCCAGGTTGCTTCATGACATGGCTGAAGGGCTTCTTCTTTCTCCATCGCAATGCTTAGGTGGCTACATGAATTTGGATGACATGGGAACCGATGCTGATGTCAAATTGTGGAATTTCTCCATTTAA

> PmCBF4

ATGGACGTGTCTGAACTTTCCGACTCGGTCGACCAGCCCGAGTCGAGTTCTTTGTCCGACGCCAGCGTCACGACTCGGGGGCCTTCTTTGTCCGACGGGGAAGTCATATTGGCGTCAAGCCGGCCGAAGAAGCGAGCCGGGAGGAGGGTTTTCAAGGAGACGAGGCACCCGGTTTATAGGGGTGTGAGGAGGAGGAACAATGACAAGTGGGTTTGTGAAATGAGAGAGCCCAACAAGAAGAAGTCCAGGATATGGCTCGGGACTTATCCGACGGCTGAGATGGCTGCTCGTGCCCATGACGTGGCGGCATTGGCGTTTAGAGGGAAGCATGCCTGCCTCAACTTTGCTGACTCCGCGTGGAGGCTGCCAGTGCCGGCTTCCATGGATCCCATGGATATTCGAAGGGCGGCCGCGGAGGCAGCTGAGGGGTTTAGGCCGGCGGAGTTTGGTGGATTGTGCAACGGCAGCAGTGATGAGAAGGAGAGAATGGTGGTGCAGGTGGAAGAGGAGAACAAGAAGGGTAGTGTGAACTTGGAAAGAAGCAGAAGCTTGAGCTTGTCCTATTGGGATGAGGAGGAAGCGTTTGACATGCCCAGGTTGCTTCATGACATGGCTGAAGGGCTTCTTCTTTCTCCACCGCAACCCTTAGGCAGCGACACGAATTTGGATGACATGGGTACCGATGCTGATATCAAATTGTGGAGTTTCTCCATTTAA

> PmCBF5

ATGGACACGATCTTCTCTCAGCTTTCTGACTCGGCCGACCAGCCCAAGTCGAGTTCGTCATCCGACGCAAGCGTGACCACCCTACGCACTTCGGACGTCATACTGGCGTCGAGCAGGCCGAAGAAGCGCGCGGGAAGGAGGGTTTTCAAAGAGACGAGGCATCCGGTTTATAGGGGTGTAAGGAGTAGGGACAACAACAAGTGGGTGTGTGAGTTGAGACAGCCCAACAAGAAGAAGTCCGGGATTTGGCTCGGGACCTATCCTACGGCTGAGATGGCTGCTCGTGCCCATGACGTGGCGGCATTGGCTTTTAAAGGGAAGCTTGCCTGCCTCAACTTTGCTGACTCCGGTTGGAGGCTGCCGGTGGCGGCATCCATGGACTCCACGGATATCCAGAGGGCAGCTGCGGAGGCCGCTGAAGGGTTCAGGCCAGTGGAGTTCGGTGGAGTTTTCAGCGACAGCAGTGATGAGAAGGAGAGAACGGTTGTGGTGGAAGAGAAGAAGAAGAAGCAGGCTATTGTGGATATGGGAAAAAGCTGCGGCAGATTAAACTTGTTTTATTCGGATGAGGAGGAAATGTTTGATATGCCAAGGTTGATTGACAACATGGCTGAAGGGCTTCTTCTTTCTCCACCGCAATGCTTAGCTGGCTACTTGAATTGGGATGACATGGAAACTGAAGCTGATCCCAAGTTGTGGAGTTTCTCTATCTGA

> PmCBF6

ATGGCTGCTCGTGCCCATGACGTGGCGGCATTGGCTTTTAAAGGGAAGCTTGCCTGCCTCAACTTTGCTGACTCCGGTTGGAGGCTGCCGGTGGCGGCATCCATGGACTCCACGGATATCCAGAGGGCAGCTGCGGAGGCCGCTGAAGGGTTCAGGCCAGTGGAGTTCGGTGGAGTTTTCAGCGACAGCAGTGATGAGAAGGAGAGAACGGTTGTGGTGGAAGAGAAGAAGAAGAAGCAGGCTATTGTGGATATGGGAAAAAGCTGCGGCAGATTAAACTTGTTTTATTCGGATGAGGAGGAAATGTTTGATATGCCAAGGTTGATTGACAACATGGCTGAAGGGCTTCTTCTTTCTCCACCGCAATGCTTAGCTGGCTACTTGAATTGGGATGACATGGAAACTGAAGCTGATCCCAAGTTGTGGAGTTTCTCTATCTGA

>PmDAM1

ATGAAAATGATGAGGGAGAAGATCAAGATCAAGAAGATTGACAACTTGCCTGCAAGGCAAGTGACCTTCTCAAAGAGGAGGAGAGGGATCTTCAAGAAAGCTGCAGAGTTATCTGTTCTGTGTGAATCTGAGGTGGCAGTTGTCATCTTTTCTGCTACTGGCAAGCTTTTTGATTATTCAAGCTCAAGTATGAAGGATGTTATTGAAAGGTACCAAGCGCACATAAATGGTGGTGAAAAATTTAACGAACGGTCTATTGAGTTGCAGCCAGAGTATGAAAACCACATCAGATTGAGCAAGGAACTTAAGGAGAAGAGCCGCCAGCTGAGGCAGATGAAAGGAGAGGATCTTGAAGAGCTGAATTTTGATGAGTTGCAGAAGTTAGAACAACTGGTGGATGCAAGCCTTGGCCGTGTGATTGAAACTAAGGACGAACGGATTATGAGTGAGATTATGGCACTTGAAAGAAAGAGAGCTGAGCTTGTAAAAGCCAACAAACAGCTAAGGCAGAGGATGTTATTCAGAGGAAATATTGGACCTGAGCTTATGAAGCCGGAGAGGTTGAATAATAATTTTGGTGGTGGAGGAGAAGAAGAAGGTATGTCATCTGAATCTGCTACCTCCACCACCTGCAACAGTGCTCCGAGTCTCTCTCTTGAAGATGACTCCGACGACGTCACTTTATCTCTCAAACTGGGGCTTCCATAA

>PmDAM2

ATGGTGAAGACGATGAGGAAGAAGATCAAGATCAAGAAGATTGACTACTTGCCTGCAAGGCAGGTGACCTTCTCAAAGAGGAGGAGAGGGATCTTCAAAAAAGCTGAGGAGCTATCTGTTCTGTGTGAATCTGAGGTGGCAGTTGTCATCTTTTCTGCTACTGGCAAGCTTTTTGATTATTCAAGCTCAAGTACAAAGAATGTTGTTGAAAGGTATAAAGCGCACACAAATGGTGTCGAAAAATCGGACGAACTGTCTGTTGAGCTGCAGCTAGAAATTGAAAACCAGATCAGATTGAACAAGGAACTTGCGGAGAAGAGCCGCCAGCTGAGGCAGATGAGAGGAGAGGATCTTGAAGAGCTGAATATTGATGAGTTGCAGAAGTTAGAACAACTGGTGGAGGCAAGCCTTGGCCGTGTGATTGAAACTAAGGAAGAACTGATTATGAGTGAGATTATGGCACTTGAAAGAAAGGGAGCTGAGCTGGTAGAAGCCAACAACCAGCTACGGCAGAGGATGGTGATGTTATCCAGAGGAAATATTGGACCTGGGCTTACGGAGCCGGAGAGGTTCATTAATAATATTGGAGATGGAGGAGAAGAAGGCATGTCATCTGAATCTGCCACAAATGCAACCATCAGCAGCTGCAGCAGTGGTCTCAGTCTCTCTCTTGAAGATGACTGCTCAGACGTCACTTTAGCTCTCAAACTGGGGCTTCCCTAA

>PmDAM3

ATGATGAGGAAGAAGATCAAGATCAAGAAGATTGATTGCTTGCCTGCAAGGCAGGTGACTTTCTCAAAGAGGAGAAGAGGGATCTTCAAGAAAGCTGCAGAGCTATCTGTTCTGTGTGAATCTAAGGTGGCAGTTGTCATATTTTCTGCTACTGGCAAGCTTTTTGATTATTCAAGCTCAAGTATCAAGGATGTTATTGAAAGCTACAAAGCGCACAAAAATGGTGTCAAAAAATCGGACGAACCGTCTGTTGAGCTACAGCTAGAGAATGAAAATCACATCGGATTGAGCAAGGAACTGGAGGAGAAGAGCCATCAGCTGAGGCAGATGAAAGCAGAGGATCTTGACGAGCTGAATTTTGATGAGTTGCAGAAGTTAGAACAACTGGTGGACACAAGCCTTAGCCGTGTGATTGAAACAAAGGAAGAACTGAGAATGAGTGAGATTATGGCACTTGAAAGAAAGGGAGCTGAGCTGGTAGAAGCCAACAACCAGCTAAAGCAGACGATGGTGATGTTATCCGGAGGAAATACTGGACCTACGCTTATGGATCCGGAGAGGTTGAATGATAATGTCGGAGGTGGAGGAGAAGAAGAAGGCATGTCATCTGAATCTGCTATCTCCACCACCTGCAACAGTGCTCTCAGTCTCTCCATTGGAGATGACTCCGACGACGTCACTTTATCTCTCGAACTGGGGCTTCCTTAA

>PmDAM4

ATGATGAGGAAGAAGATCAAGATCAAGAAGATTGACTACCTGCCAGCAAGGCAGGTGACATTCTCAAAGAGGAGAAGAGGGATCTTCAAGAAAGCTGCAGAGCTATCTGTTCTGTGTGAATCTGAGGTGGCAGTTGTCATCTTTTCTGCTACTGGCAAGCTTTTTGATTATTCAAGCTCAAGTATCAAGGATGTTATTGAAAGGTACAAAGCGCGCACAAATGGTGTCGAAAAATCGGATGAACAGTCTCTTGAGCTGCAGCTGGAGAATGAAAACCGCATCAAACTCAGTACGGAACTCGAGGAGAAGAACCGCCAGCTGAGGCGGATGAAAGGTGAGGATCTTGAAGAGCTGGATCTGGATGAGTTGCTGAAGTTGGAACAACTGGTGGAAGCAACCCTTGTCCGTGTGATGGAAACTAAGGAAGAACTGATTATGAGTGATATTGTGGCACTTGATAAAAAGGGAACTGAGCTGGTAGAAGGCAACAATCAGATGGTGATGTTAAGGGACAGGATGGTGATGTTATCCAAAAGAAGTACCGGACCTGCGCTTATGGAGCCATCTGACTCTGCTACCTCCACCAGCTGCAACAGTGCTCTGAGTCTTTCTCTTGAAGATGAATGCTCCGACGACGCCATTTTATCTCTCGAACTGGGGCGTTCCTAA

>PmDAM5

ATGATGAATAAGATCAAGATCAAGAAGATTGACTACTTGCCTGCAAGGCAGGTGACCTTCTCAAAAAGGAGAAGAGGGCTCTTCAAGAAAGCTGCAGAGCTATCTGTTCTGTGTGAATCTGAGGTGGCAGTTGTCATCTTTTCTGCCACTGGCAAGCTTTTTGATTATTCAAGCTCAAGTACCAAGGATGTTATTGAAAGGTACAACGCAGACATGAATGGTGTCGAAAAATCGAACAATCAAGAGATTGAGCTGCAGCTGGAGAATGAAAACCACATCAAACTGAGTAAGGAACTCGAGAAGACGAGCCACCAGCTGAGGCAGATGAAAGGTGAGGATCTTGAAGGGCTGAATCTGGATGAGTTGCTGAAGTTGGAACAACTGGTGGAAGCAAGCCTTGGCCGTGTCATGGAAACTAAGGAAGAGCTGATTAAGAGTGAGATTATGGAACTCGAAAGAAAGGGAGCTGAGCTAGTTGAAGCCAACAGCCAGCTAAGGCAGACGATGGTGATGTTATCCGGAGGAAATACTGGACCTGCGCTTATGGATCCGGAGAGGCTGAATAATAATATTGAAGGTGGAGGAGAAGAAGAAGGCATGTCAGCTGAATCTGCTATCTCCACCACCTGCAACAGTGCTGTCAGTCTCTCTCTTGAAGATGACTCCTCCGATGAGGTCACTTTGTCTCTCAAACTGGGGCGTTAA

>PmDAM6

ATGGTGAAAATGATGAGGGAGAAGATCAAGATCAAGAAGATTGACTACCTGCCAGCAAGGCAGGTTACCTTTTCAAAGAGAAGAAGAGGGCTCTTCAAGAAAGCTGCAGAGCTATCGGTTCTGTGTGAATCTGAGGTGGCTGTCGTCATCTTTTCTGCCACTGACAAGCTCTTTCATTATTCAAGCTCAAGTACCGAGGATGTTATTGAAAGGTACAAAGCGCACACAGGTGGTGCCGAAAAATCAGACAAACAGTTTCTTGAGCTGCAACTGGAGAATGAAAACAACATCAAACTGAGTAAGGAACTCGAGGAGAAGAGCCGCCAGCTGAGGCAGATGAAAGGTGAGGATCTTGAAGGGCTGAATCTGGATGAGCTGCTGAAGTTAGAACAAGTGGTGGAAGCAAGCCTTGGCCGTGTGATAGAAACTAAGGAAGAGCTGATTATGAGTGCGATTATGGCACTGGAGAAAAAGGGAGCTGAGCTGGTAGAAACCAACAACCAGTTAAGGCATAGGATGGTGATGTTATCCGGAGGAAATACTGGACCTGCGTTTGTGGAGCCGGAGACGTTGATTACTAATGTTGGAGGTGGAGGACGAGAAGACGACATGTCATCTGAATCTGCCGTAATTGCCACCTCCACCAGCTGCAACAGTGCTTTCAGTCTCTCTCTTGAAGATGACTGCTCCGATGTCACTTTATCTCTCAAACTGGGGCTTCCCTAG

**Supplementary Data S2.** The 2kb up-stream promoter sequence of *PmDAM6*.

> proDAM6

AAAAGAAAAACTACGAATTGCTGATCCCA**CCGAC**ATGTTTTTTAATATAGAAATATTGACAATTCAAAAAAGTCATCACGAATTTCAGATAAATCAAAAACAATTGCACGATGATTTATTTCCCAGCAACTTCAATATTTTCAGTAATATAGTATAAGAGCTCATTTGGGCCCAACTGTTTTCACCTATTTACTACACCAGAGCTTCTACAATGAGGAGTTTAGAGTGAGATGTTTATTTGAGGGGTTTAATCCCACTATCAAACTATTTAAAATGTTTCAGTTCATTGATTTCCTTTCATTTGTTGATGGTGGGATTGAACCCCTCAAATAAGCTCATCACTCTAAACACTTCATTAGAGCCTTTCCGTTTGCGTACTAACCCTCCCAATTCGATTGAAGATGTTTTAGGGGTTTGTAGATCAAGTTTCTTTTGAGGTTTCATTATTATGTATTTGGGCCATTTCATTTTTTGTGTTTATTTAAATTGAAAGAAGTGACTTTCACTGAAATTTCCCATTTTTTAAATTATATTTTGTTATAGAAAATAATGGATGGCTTATGTGAATTAACGTCATTATTATTGCGTAGTATGGCAATCTAAATCAAATTAATTTAAACATTTTTTGTATAAAAATAAAAATAATTAACTTTCCCGCATTGTTCTATGAAAATTACAGAAAGCTTCAGTCGAGGAATCTCAACACTGAAAGGGGCTGAAAACGTAGGGGAGAATTTATTCCACACTCCCGCTATGACAGCATAACCTGCATAATACAAGTTAAAACATCCCCACAAATAAATGTTCGTGCACAGTTGTATTCATGGTATTTCATTTATTCCTCACATTTTAAATAAATTTACTAATCATTTTGTACGTATTTTTTCCGAATTTCAGGCGTTAAGAAGTGGAGAAAGATGAAAGTTTACTCTTCTTATAACTAAATAAAGAAAGTCGAGGTATTGTAAGATATCAGTGATTATGTGTCTTAACTCGACATAACATTGAAAAATATCAAAGGGAGGGTGACTCTGTGTTGTATCTAAACCTATGCGTTACATGCAGCTTGAAATTGCTCTGACTTGGTATTGTATTTAAAACTTTGTATTAATGCAGCTTGCATGGTGAATTATGTTGATTTTGAATTTGCTTGGGTTATCAATGTCACGGCGAGACACGTACTGTTTGCGTGGATAAAAATGAAGTGGGGGAAGGAACCTGAGCAAGATATAGACAGCCACGTGTCGCATGGTGGGCCAATCAAGTGACATGACAGCGAATGACCTGTCTCCAT**CCGAC**TCCACATAAAAATAAAATTTATTATTTCTTTTGTACACGTAGTAAGAATTAAA**CCGAC**AACAAACTAAACTTTAGATACTTCTGACCTAAAAAAACATAGCTTAGATATGGACAAAATCAATCACAAAAAGTAAGATGCTGCGGTTTGCGCTGTCGCATTACAAGATCCACGGTGGAGATTGCATGCATCAACGTTAAGGTGCATCTGTCCCCGTCGATTTGCTCAGGTTTCGTTGTTTTCCCAGAAGAGACTGAAGTAAAGCAGAGGATAATAAAAATAGATGTTTTCCATAAAAAGGAAAATTTCCTTTCTTGAACAACTAGCCACCAACAGCAGCCGCCCTTTATCTCTCTAATTCTCTTAGTTTTTCTGGACAGACCAAAACTTCAGACCGGGCTGAAACCCC**CCGAC**AAAGGTAAACATTAAAGACAGAGAGGAAAACCCAGAGATTAATTGATTAAATTAATTAGGGTTCTTCCTCTTCTTCTTCGTCTTCTTCTTCTTCTTCTTCTTTTTGTTGTGAACTTGTAACCTATTTTGGTTGGTGGGTTTTTCTGGGTTTTGTTCATTTAGATCTGGGGACCATTAAAAGGTTAAATTAATTGAGAAGGAACCCAGAAAATATTGTGTAGTTTTTTAGTGTGTGAAGATAATAATATATGTGAAAAGTGGTTGGTTTGAATTTTTTTGAAGGGAA

**Supplementary Data S3.** The sequences of pAbAi-genes.

>pAbAi-1-1 (including M2 and M3)

ACCTGTCTCCAT**CCGAC**TCCACATAAAAATAAAATTTATTATTTCTTTTGTACACGTAGTAAGAATTAAA**CCGAC**AACAAACTAAAACCTGTCTCCAT**CCGAC**TCCACATAAAAATAAAATTTATTATTTCTTTTGTACACGTAGTAAGAATTAAA**CCGAC**AACAAACTAAA

>pAbAi-1-3 (including M2 and M3)

ACCTGTCTCCAT**CCGAC**TCCACATAAAAATAAAATTTATTATTTCTTTTGTACACGTAGTAAGAATTAAA**CCGAC**AACAAACTAAA

>pAbAi-2-1 (including M4)

AATTCTCTTAGTTTTTCTGGACAGACCAAAACTTCAGACCGGGCTGAAACCCC**CCGAC**AAAGGTAAACATTAATTCTCTTAGTTTTTCTGGACAGACCAAAACTTCAGACCGGGCTGAAACCCC**CCGAC**AAAGGTAAACATT

>pAbAi-2-3 (including M4)

AATTCTCTTAGTTTTTCTGGACAGACCAAAACTTCAGACCGGGCTGAAACCCC**CCGAC**AAAGGTAAACATT

>pAbAi-3-1 (including M1)

AAAAGAAAAACTACGAATTGCTGATCCCA**CCGAC**ATGTTTTTAAAAAAGAAAAACTACGAATTGCTGATCCCA**CCGAC**ATGTTTTTAA

>pAbAi-3-3 (including M1)

AAAAGAAAAACTACGAATTGCTGATCCCA**CCGAC**ATGTTTTTAA
